# Single-cell classification based on label-free high-resolution optical data of cell adhesion kinetics

**DOI:** 10.1038/s41598-024-61257-2

**Published:** 2024-05-16

**Authors:** Kinga Dora Kovacs, Balint Beres, Nicolett Kanyo, Balint Szabó, Beatrix Peter, Szilvia Bősze, Inna Szekacs, Robert Horvath

**Affiliations:** 1 Nanobiosensorics Laboratory, Institute of Technical Physics and Materials Science MFA, HUN-REN Centre for Energy Research, Konkoly-Thege út 29-33, 1121 Budapest, Hungary; 2 Department of Biological Physics, Eötvös University, Budapest, Hungary; 3 Department of Automation and Applied Informatics, Faculty of Electrical Engineering and Informatics, Budapest University of Technology and Economics, Műegyetem Rkp. 3., 1111 Budapest, Hungary; 4 HUN-REN-ELTE Research Group of Peptide Chemistry, Hungarian Research Network, Eötvös Loránd University, 1117 Budapest, Hungary; 5 Cellsorter Kft., Budapest, Hungary

**Keywords:** Motility, Nanoscale biophysics, Cancer, Cell biology, Optics and photonics

## Abstract

Selecting and isolating various cell types is a critical procedure in many applications, including immune therapy, regenerative medicine, and cancer research. Usually, these selection processes involve some labeling or another invasive step potentially affecting cellular functionality or damaging the cell. In the current proof of principle study, we first introduce an optical biosensor-based method capable of classification between healthy and numerous cancerous cell types in a label-free setup. We present high classification accuracy based on the monitored single-cell adhesion kinetic signals. We developed a high-throughput data processing pipeline to build a benchmark database of ~ 4500 single-cell adhesion measurements of a normal preosteoblast (MC3T3-E1) and various cancer (HeLa, LCLC-103H, MDA-MB-231, MCF-7) cell types. Several datasets were used with different cell-type selections to test the performance of deep learning-based classification models, reaching above 70–80% depending on the classification task. Beyond testing these models, we aimed to draw interpretable biological insights from their results; thus, we applied a deep neural network visualization method (grad-CAM) to reveal the basis on which these complex models made their decisions. Our proof-of-concept work demonstrated the success of a deep neural network using merely label-free adhesion kinetic data to classify single mammalian cells into different cell types. We propose our method for label-free single-cell profiling and in vitro cancer research involving adhesion. The employed label-free measurement is noninvasive and does not affect cellular functionality. Therefore, it could also be adapted for applications where the selected cells need further processing, such as immune therapy and regenerative medicine.

## Introduction

During evolution, eukaryotes developed spatially separated organelles for performing various functions, and just like that, multicellular organisms arranged special cell types into tissues and organs in a highly organized manner. To form these complex structures, cells need to connect to each other and do so in a way that fulfills the needs of different tissue types^1^.

The various cellular connections are realized by four main cell adhesion molecules: the family of integrins, cadherins, immunoglobulins, and selectins. The predominant role in cell–matrix adhesion belongs to integrins. This heterodimeric transmembrane receptor anchors the cell to the extracellular matrix (ECM) by binding it to the actin cytoskeleton at the intracellular terminal. Integrins are not only capable of binding to the ECM, but exhibit signaling properties^2^ as well. Each integrin comprises an alpha and beta subunit and shows a significant variance in their composition. There have been 18 alpha and 8 beta subunit variants described so far in mammals^3^. This combinatoric diversity is a common phenomenon in molecular biology, resulting in the capability to realize innumerable functions with a limited number of molecular modules. It potentiates the many roles of integrins in seemingly distant biological processes, such as embryonic development, inflammatory responses, or tumor metastasis formation^4,5^. Cell adhesion can be modulated by the expression of adhesion receptors and their conformational changes, receptor trafficking in the plasma membrane, integrin clustering, receptor–cytoskeleton interactions, and by the glycocalyx. The glycocalyx—a carbohydrate-enriched sugar coating—regulates the strength and speed of cancer cell adhesion^6^.

Integrin expression level is altered in cancer cells. Therefore, integrins are considered potential targets for cancer therapy^7^. The distinctive mechanism of tumor cell adhesion from that of normal cells underlies the increased migration, invasion, proliferation, and survival of cancer cells.

Cellular heterogeneity appears in multicellular organisms even in ostensibly homogeneous tissues. Due to advances in genomics, it is now possible to sequence the genetic material of single cells to a level that enables whole-genome sequencing, providing extremely rich data^8,9^.

If the goal is to measure/manipulate single cells in their quasi-native state, label-free and minimally invasive techniques are preferably applied. Among a wide range of techniques, one such notable example is the robotic fluidic force microscopy (FluidFM) system that enables not only single-cell level measurements but interactions as well, e.g. extraction or infusion of cellular material, opening the field of single-cell secretomics^10–12^. On the bleeding edge of today's cell biology, high-throughput data acquisition tools are paired with machine learning algorithms for analyzing the vast amount of data and drawing relevant conclusions from it.

The majority of traditional methods investigating cellular processes at the molecular level require labeling, however, the label molecules can interact with the biological system in an unintended way, modifying its native state. In contrast to that, label-free techniques have been developed, which enables measurements that have practically no effect on the studied processes. These can be calorimetric, electrochemical, piezoelectric, or optical biosensors^13–20^.

Optical biosensors measure refractive index changes within a 100–150 nm range from the sensor surface, monitoring the resonance peak as the function of incident angle (optical waveguide lightmode spectroscopy (OWLS), surface plasmon resonance (SPR)) or resonant wavelength/phase shift (resonant waveguide grating (RWG), grating-coupled interferometry (GCI)). Epic Cardio (Corning Inc.) is a high-throughput RWG optical biosensor compatible with standard 384-well microplate format (supplemented with a glass insert containing the resonant waveguide gratings), suited for high-throughput biological experimenting^23,24^.

If cell adhesion is monitored on an optical biosensor (in particular RWG sensors), the estimated 150 nm deep evanescent field overlaps with the extracellular matrix (modeled as a surface coating, 10–80 nm), the cell membrane with its associated proteins such as integrin and vinculin (10–20 nm), and part of the actin cytoskeleton itself^11^. Thus, the information collected by the RWG biosensor from the cell-substrate area highly indicates the adhesion process. The RWG signal was recently calibrated to adhesion force using a combined RWG—robotic fluidic force microscopy (FluidFM) setup^11^. Notably, the kinetics of processes can be studied with high resolution and an excellent signal-to-noise ratio. From the kinetic data, valuable molecular scale information can be obtained, such as the binding constants of receptor-ligand interactions^6,25–27^.

Label-free monitoring techniques are on the rise in both basic and applied research, but their fusion with cutting-edge machine-learning techniques is still lagging behind. Generally, classification tasks can be split into two main parts: finding a suitable representation of the data by some transformations –usually referred to as features– and creating a classifier that can discriminate these features. In the case of kinetic data (time-series input), general features can be identified by Fourier Transform, Wavelet Transforms, or Symbolic Mappings^28^. Another possibility is to fit a theoretical model to our experimental observations and use the model’s relevant parameters as features for classification. However, for most of the studied systems, the lack of these readily available models is the very reason we choose more general approaches.

Single-cell classification with machine learning has emerged as a powerful tool in biological research, enabling the categorization of individual cells based on their unique features. By leveraging sophisticated algorithms and high-dimensional data analysis techniques, machine learning models can discern subtle differences between cell types, aiding in the identification of rare cell populations and the understanding of complex biological systems^52^. These models utilize various types of data, including gene expression levels, protein abundances, and cellular morphology and motility^49,50,53,54^ and spectral information^51^ to accurately classify cells into distinct categories. The application of single-cell classification with machine learning holds immense promise for advancing our understanding of cellular heterogeneity, disease mechanisms, and personalized medicine.

A prominent difference in the workings of deep learning-based classifiers vs. feature-based ones is that the former methods are (in most cases) jointly trained for feature extraction and classification in an end-to-end manner, to learn from latent representations of the input data. Thus, the representations are not hard-coded or user-defined but rather formed optimally to the specific input dataset and classification task. For time-series data processing, the most common models are based on convolutional neural networks (CNNs)^29,30^ or recurrent neural networks (RNNs) architectures.

Despite the recent general trend of moving from “traditional” approaches towards deep learning-based methods reaching human-level performance in various tasks such as computer vision, label-free biosensor data analysis lacks proper AI solutions^31^.

Usually, the more complex the theoretical model, the harder it is to draw intuitive conclusions from it. On the other hand, simple models may not grasp the fine details of the system, thus providing false findings. Machine learning models are trained to perform a specific task based on a given input dataset. Because of this inherent black box model nature of the deep learning-based models, even though they reach a high level of accuracy in a specific task they may not provide a deeper understanding of the underlying phenomenon. However, depending on model architectures, there are several methods specifically tailored to unravel these representations providing useful insights^32^. In the case of CNNs, two methods have been developed: one tries to understand the model's decisions by mapping the output (and intermediate variables) to the input space, giving local explanations (grad-CAM), and the other with the global interpretation of the processing layer elements. RNNs are used for sequential data processing, and although the model family has outstanding performance in this field, only a few methods have been proposed to interpret their decision rules^32^.

In the present work, we introduce a method for RWG-based label-free cell classification leveraged by several deep learning-based benchmark models previously used for classification tasks for a diverse array of datasets. The basis of selection is the different adhesion kinetic signals of five types of cancer cells recorded by the biosensor. The employed label-free measurement is noninvasive and does not affect cellular functionality. Consequently, it could also be adapted for applications where the selected cells must be processed further (e.g. immune therapy and regenerative medicine).

## Materials and methods

All chemicals and reagents were obtained from Sigma-Aldrich Chemie GmbH (Schelldorf, Germany), unless stated otherwise.

### Cell cultures and cell assays

HeLa cells, a cervical cancer cell line (ECACC 9302113) was maintained in Dulbecco’s modified Eagle’s medium (DMEM, Gibco) supplemented with 10% fetal bovine serum (FBS, Biowest SAS, France), 4 mM l-glutamine, 100 U/ml penicillin and 100 μg/ml streptomycin solution.

The osteoblastic cell line MC3T3-E1 (ECACC 99072810) was maintained in α-modified minimal essential medium supplemented with 10% FBS (Biowest SAS, France), 2 mM l-glutamine, 100 U/ml penicillin and 100 μg/ml streptomycin solution.

The LCLC-103H (large cell lung carcinoma cell line, ACC384) and two breast cancer cell lines, MDA-MB-231 (ECACC 92020424) and MCF-7 (ECACC 86012803), were maintained in DMEM supplemented with 10% FBS (Biowest SAS, France), 1% non-essential amino acids, 1 mM sodium pyruvate, and 100 U/ml penicillin and 100 μg/ml streptomycin solution.

Cells were cultured in a humidified atmosphere containing 5% CO_2_ at 37 °C.

For the experiments, cells were removed from the tissue culture dishes using a standard protocol with 0.05% (w/v) trypsin and 0.02% (w/v) EDTA solution.

The harvested cells were centrifuged at 200×*g* for 5 min and the cell pellet was re-suspended in assay buffer (20 mM 2-[4-(2-hydroxyethyl)piperazin-1-yl]ethanesulfonic acid (HEPES) in Hank’s balanced salt solution (HBSS), pH 7.4). The centrifugation was repeated two times to remove the cell culture media completely. Cells were then counted in a hemocytometer and diluted to a final cell density of 200 cells in 25 µl of HEPES-HBSS solution.

The measurements were carried out at room temperature. 25 µl assay buffer was added to 12 wells in a fibronectin-coated (10 µl of 30 µg/ml fibronectin solution in each well incubated for 1 h then washed 3 times with MQ water) Epic 384-well cell assay microplate (purschased with fibronectin-coating from Corning Incorporated, Corning, NY, USA). The baseline was recorded for 90 min before 25 µl cell suspension was added to each well and the cell adhesion was measured for up to 3 h.

The constructed single-cell database contains the data of 13 independent experiments carried out with the above-described protocols.

### Single-cell resonant waveguide grating (RWG) sensor

Epic Cardio (Corning Inc., USA) is an optical label-free biosensor leveraging the RWG (Resonant Waveguide Grating) technology. The primary sensor input is a near-monochromatic infrared laser with a tunable wavelength in the range of 825–840 nm. In the waveguide, only a specific wavelength of light that satisfies the resonance condition can be incoupled, which condition is dependent on the refractive index of close vicinity (100–150 nm) of the sensor surface. The reflected light then reaches a fast CMOS (Complementary Metal–Oxide–Semiconductor) camera with a resolution of 80 × 80 pixels, that can record a scan of the entire wavelength range with step size of 0.25 pm under 3 s. To denoise the measurements multiple scans are recorded and averaged in the output. The physical dimensions of a single sensor are 2 × 2 mm, and the instrument has a 25 µm lateral spatial resolution, making it suitable for single-cell detection, a single cell usually occupying 1–4 pixels. Of note, using much higher lateral resolution would limit the number of cells measured to 1–5 instead of the hundreds of cells we can monitor parallel.

### Cardio alpha—custom-developed data analysis software

As the single-cell RWG biosensor can record the raw wavelength shift (WS) of up to 1200 single cells in a session, a suitable automatized data preprocessing pipeline was created, to satisfy the processing needs of large-scale single-cell database building.

The raw measurements are produced in each timestep in the form of 12 concatenated 80 × 80 pixel matrices, holding the current wavelength/wavelength-shift values. Our software first reads this file and transforms it into a single “video” matrix, with 2 spatial (80 × 80 for a whole well’s sensor area) and 1 temporal dimension (length of the measurement). Because the measurements could contain artifacts and baseline drift, a global background correction is performed which selects pixels of the sensor that do not correspond to cell signals and subtracts the average of these from the whole measurement. This method can robustly eliminate non-cell-related signals (noise or drift) from the measurement data.

As single cells showing distinct peaks on WS maps, their detection can be formulated as a 2D local maxima finding problem. To separate relevant cells from noise in the background, the user can define a minimal detection threshold below which all detected local maxima are neglected. After detection is done, the sensor area is partitioned by Voronoi-tessellation defined by the cell peaks, thus assigning a region of interest (ROI) for each single cell. The maximum signal value within these ROIs is monitored and assigned to the cell’s maxWS signal.

### Data pre-processing

After the measurement, single cells were identified using bounded peak local maxima search in the 80 × 80 pixel cardio images. The maximum pixel signals were exported from each identified cell. Since these generally have a small offset, due to the global drift correction, the signals were corrected by subtracting the first measured value from the whole single-cell signal, pushing it to zero. As the addition of cell solution introduces a measurement-specific artifact due to the slightly different temperature/consistency of the buffer solution, recordings were corrected with these batch effects. Afterward, we cut the signals to uniform lengths to produce a suitable input for the classification models. To map the optimal measurement length for the classification task, we created datasets of 30, 60, 90, 120, and 150-min intervals.

To check the classification performance based on the cell types, several datasets were constituted based on different cell type selections. The same signals are contained across the different datasets. This should change the classification performance, because of the change in the overlapping of the different cell distributions.

To compensate for class imbalance in our dataset, we used Synthetic Minority Over-sampling Technique (SMOTE)^42,43^ based data augmentation to oversample the training signals, so that each class has a matching sample count to the majority class’. Instead of using standard Random Oversampling (ROS), where the oversampling happens by randomly repeating samples from the original set, SMOTE synthesizes samples by taking a random point for between a selected sample and its k-nearest neighbors(k-NNs) for each timestep^48^. Table 1 summarizes the number of single cells used for classification.

**Table 1:** The datasets created from the measurement data show how many individual cells took part in the classification.

Name	30 min	60 min	90 min	120 min	150 min
HeLa	909	909	909	909	909
LCLC-103H	376	376	376	376	376
MC3T3-E1	767	767	767	767	767
MCF-7	1000	1000	1000	349	349
MDA-MB-231	1466	1466	1466	1466	1466
Breast cancer	2466	2466	2466	1815	1815

Since there is a high variation in the signal amplitude, we standardized the datasets before the training and validation processes. Both the training and validation sets were standardized using the mean and standard deviation values of the training set. Note that this transformation erases the absolute signal magnitude information from the data, however, preliminary experiments showed that using a magnitude preserving normalization (e.g. max scaling across the whole dataset) would not result in better performance.

### Deep learning based classification

Several models were applied for classification using the Deep Learning for time-series classification library^41^ developed and maintained by Fawaz et al. It contains most of the widely used convolutional neural network-based time series classification models that have already been tested on a wide array of datasets. We used six of the available models: the Multi-Layer Perceptron (MLP), Convolutional Neural Network (CNN), Fully Convolutional Network (FCN), InceptionTime^45^, Multi-Channel Deep Convolutional Network (MCDCNN)^46^ and Residual Network (ResNet) models. All the models are implemented using TensorFlow’s Keras^47^ library. The architecture of these models are listed in Table 2. with the optimizer, loss and training hyperparameters. Since we performed several consecutive training sessions with the selected networks, we implemented an early stopping module in the existing codebase to optimize the training time.

**Table 2:** The architecture of the employed six neural networks (MLP, CNN, FCN, ResNet, MCDCNN and Inception) with the optimizers, loss functions and hyperparameters used for the training process.

Methods	Architecture						
	Layers	Conv	Norm	Pooling	Feature	Act	Reg
MLP	4	0	–	–	FC	ReLU	Dropout
CNN	4	3	–	Avg	FC	–	–
FCN	5	3	Batch	–	GAP	ReLU	–
ResNet	11	9	Batch	–	GAP	ReLU	–
MCDCNN^46^	4	2	–	Max	FC	ReLU	–
Inception^45^	7	6	Batch	Max	GAP	ReLU	–

Methods	Optimizer						
	Algorithm	Loss	Epochs	Batch	LR	Decay	
MLP	AdaDelta	CrossEntropy	500	32	0.001	0.5	
CNN	Adam	CrossEntropy	500	16	0.0001	–	
FCN	Adam	CrossEntropy	500	32	0.0001	0.5	
ResNet	Adam	CrossEntropy	500	32	0.0001	0.5	
MCDCNN^46^	SGD	CrossEntropy	500	32	0.001(0.9)	–	
Inception^45^	Adam	CrossEntropy	500	32	0.0001	0.5	

### Training

The training process was done using stratified five-fold cross-validation^44^ for all datasets. Every model was trained five times on each dataset with rotated partitioning of the training and test datasets. The train and test datasets were selected with 80–20% ratio. After partitioning, each dataset was preprocessed and augmented according to the methods discussed in the data pre-processing section. The evaluation metrics were calculated on the selected test dataset in each training iteration. The final performance was evaluated based on the statistical properties of these iteration metrics. Hyperparameter optimization was only done on the learning rate and batch size parameters of each model. The factory default loss function and the optimizer were kept for the training.

### grad-CAM visualization

Selvaraju et al. introduced grad-CAM^33^ (gradient-Class Activation Map) as an explanatory visualization technique for CNNs. This method calculates gradients of output values in different categories with respect to feature map activations of convolutional layers. The resulting gradients are globally average-pooled over the feature map to determine neuron importance (${\alpha }_{k}^{c}$). Positive weights (${\alpha }_{k}^{c}$) indicate that features on a specific map contribute to the final decision (${y}^{c}$). Deep CNN feature maps capture high-level visual concepts, retaining spatial arrangement due to convolutional operations. By calculating gradients, we gain insight into the model’s decision-making process. The final grad-CAM is formed by combining feature maps using these weights and passing through a ReLU to keep parts correlating with the output class (${y}^{c}$).

1 $${L}_{grad-CAM}^{c}=ReLU\left({\sum }_{k}{\alpha }_{k}^{c}{A}^{k}\right)$$

The map's size matches the convolutional feature map, tying localization ability to the target feature map's resolution, often decreasing with depth in deep CNNs. The last convolutional layer is typically used for grad-CAM creation.

## Results and discussion

**Figure 1:**
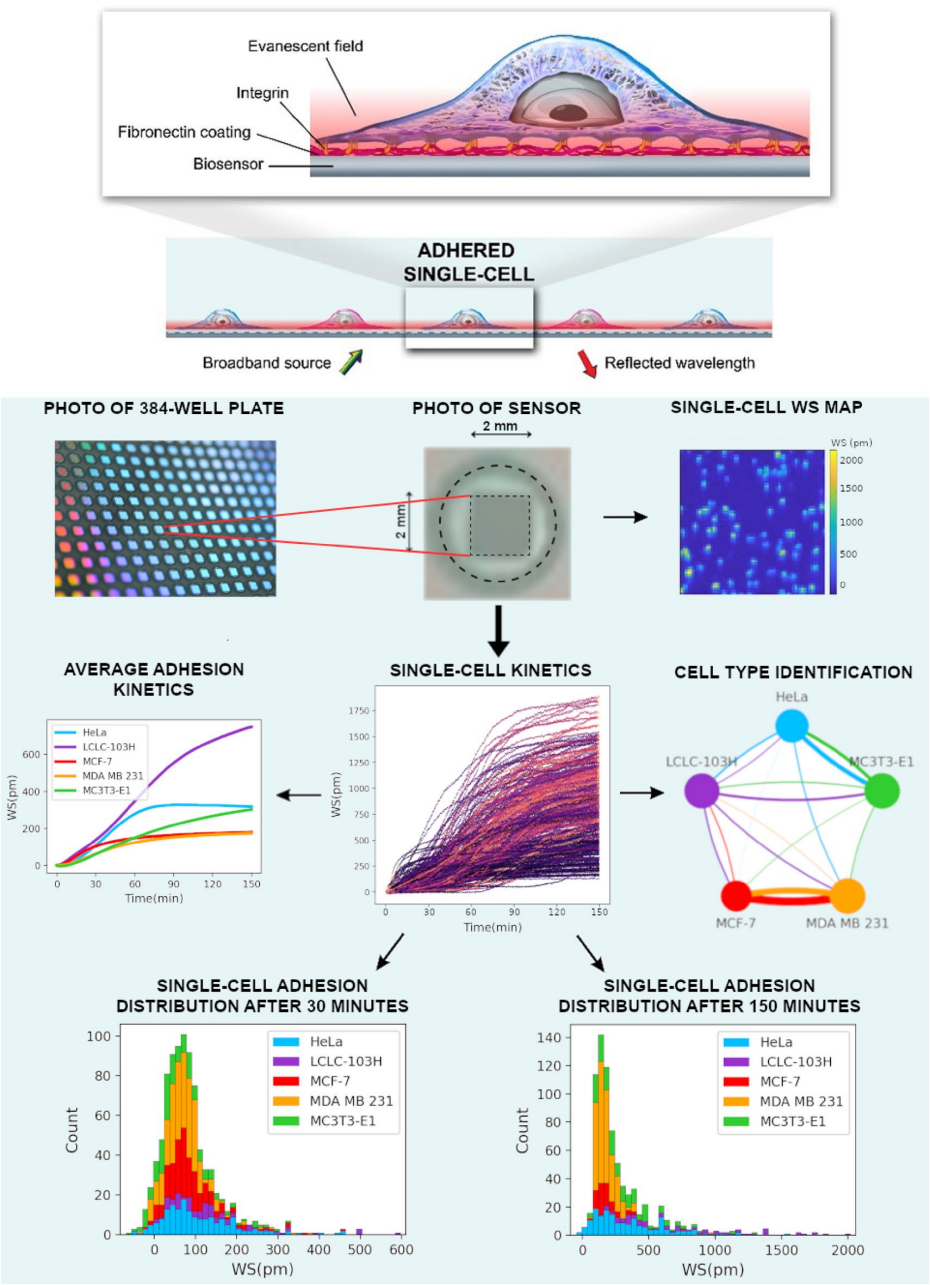
Label-free acquisition of single cell adhesion kinetics. Cells were added to a standard 384-well optical biosensor plate and functionalized with a cell adhesion-promoting coating. Representative wavelength-shift (WS) map at a given timestep of a single well in a typical single-cell RWG biosensor measurement. Peaks in the image indicate adherent cells. Single-cell signals after preprocessing. Average cell adhesion signal for every cell type. A confusion graph was obtained from the cell type classification. Distribution of cell adhesion signals at 30 (left) and 150 (right) minutes.

The measurement protocol and the workflow are summarized in Fig. 1. We measure around a hundred cells in one well of the 384-well biosensor plate, which can be identified as peaks on the single-cell WS map. After data preprocessing, we can get the single-cell kinetic data. From this, we can obtain the average adhesion kinetics for every cell type and the single-cell adhesion distributions for each time point. After classification, we can identify the cell types from the single-cell adhesion signals.

### Performance comparison of the applied networks

**Figure 2:**
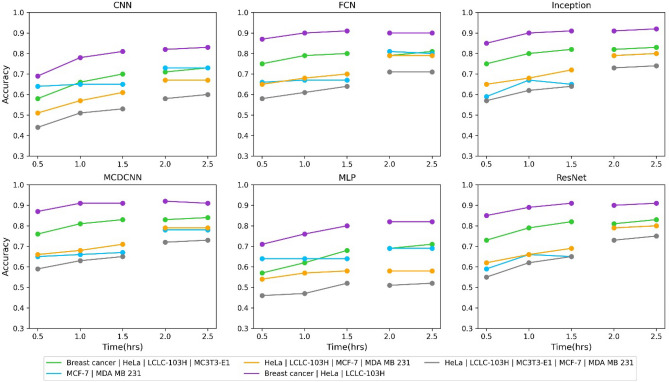
How the accuracy of the six different neural networks changes in time. The measurements were 2.5 h long, the data was evaluated every 30 min, and the accuracy was plotted for each neural network. The figure displays the mean accuracy value of the cross-validation iterations. A gap is displayed between the 1.5 and 2 h results because of the change in the sample count for the MCF-7 cell type.

To determine which neural network performed the best, we initially compared them based on their accuracy and how this accuracy changed over time. The cell adhesion was measured for 2.5 h. We were interested in understanding how the measurement time influenced accuracy. The results are depicted in Fig. 2.

For all the neural networks, accuracy notably improves over time until 1.5 h. However, after that, there is no significant improvement. However, the accuracy of the other three neural networks (FCN, ResNet, and Inception) does not exhibit an increase with measurement time.

To determine the best-performing neural network, we compared MCDCNN, ResNet, and Inception, all of which exhibited the highest accuracies previously in the following table.

In the five cases depicted in Table 3, we observed that MCDCNN was overall the highest-performing neural network. As illustrated in Fig. 2, the accuracy of MCDCNN remained nearly constant over time, indicating that a mere 30-min measurement is sufficient for cell-type classification.

**Table 3:** Comparison of the different neural networks, which previously showed the best accuracy, (ResNet, Inception, and MCDCNN) based on precision, accuracy, and recall evaluated with 90 min of measurement data.

Setup	Network	Precision	Accuracy	Recall
MCF-7|MDA-MB-231	ResNet	0.64 ± 0.01	0.65 ± 0.02	0.64 ± 0.01
	Inception	0.66 ± 0.03	0.65 ± 0.03	0.63 ± 0.02
	**MCDCNN**	**0.66 ± 0.02**	**0.67 ± 0.02**	**0.64 ± 0.02**
HeLa|LCLC-103H|MCF-7| MDA-MB-231	ResNet	0.7 ± 0.02	0.69 ± 0.01	0.68 ± 0.01
	**Inception**	**0.73 ± 0.02**	**0.72 ± 0.01**	**0.71 ± 0.01**
	MCDCNN	0.71 ± 0.01	0.71 ± 0.01	0.71 ± 0.01
Breastcancer|HeLa|LCLC-103H	ResNet	0.84 ± 0.01	0.91 ± 0.0	0.81 ± 0.03
	Inception	0.84 ± 0.02	0.91 ± 0.01	0.8 ± 0.03
	**MCDCNN**	**0.85 ± 0.02**	**0.91 ± 0.01**	**0.85 ± 0.01**
HeLa|LCLC-103H|MC3T3-E1|MCF-7|MDA-MB-231	**ResNet**	**0.66 ± 0.01**	**0.65 ± 0.01**	**0.64 ± 0.01**
	Inception	0.65 ± 0.03	0.64 ± 0.02	0.63 ± 0.02
	MCDCNN	0.65 ± 0.02	0.65 ± 0.02	0.65 ± 0.02
Breastcancer|HeLa|LCLC-103H|MC3T3-E1	ResNet	0.73 ± 0.01	0.82 ± 0.01	0.71 ± 0.01
	Inception	0.74 ± 0.03	0.82 ± 0.02	0.72 ± 0.03
	**MCDCNN**	**0.75 ± 0.02**	**0.83 ± 0.01**	**0.74 ± 0.02**

The results shown are the mean and standard deviation values for the five-fold cross-validation.

Significant values are in bold.

For further analysis, we are using 120 or 150 min of measurement data evaluated with the MCDCNN neural network.

### Breast cancer type differentiation

We were curious to know if our biosensor data were suitable for classifying two human breast cancer cell lines, both adenocarcinomas.

**Figure 3:**
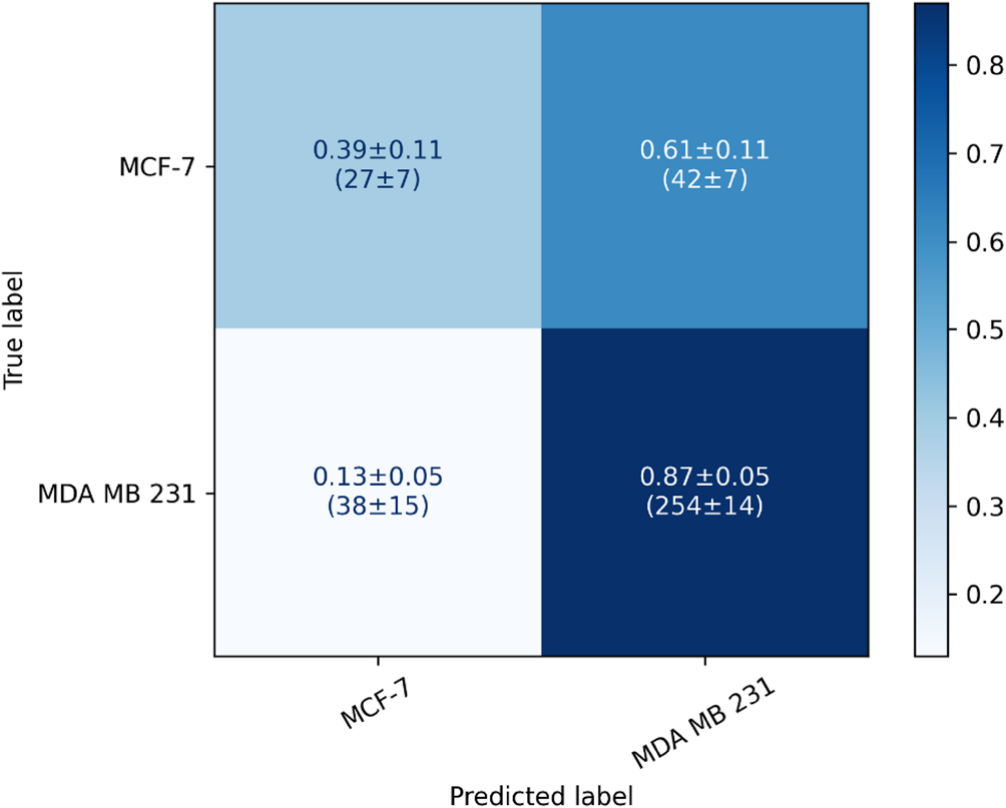
Confusion matrix calculated from the test datasets of the cross-validations (MCDCNN, 120-min) of the two breast cancer cell lines MDA-MB-231 and MCF-7.

With only those two cell lines the classification accuracy was around 67% (Table 2). Based on the confusion matrix (Fig. 3) the identification of MDA-MB-231 cells is much better than the identification of MCF-7 cells with a true positive rate of 87% and 39%. The neural network identifies 61% of MCF-7 cells as MDA-MB-231 cells.

### Cancer type differentiation

Next, we tried to distinguish between the four different cancer cell types, the two breast adenocarcinomas, a cervical adenocarcinoma, and a large cell lung carcinoma.

**Figure 4:**
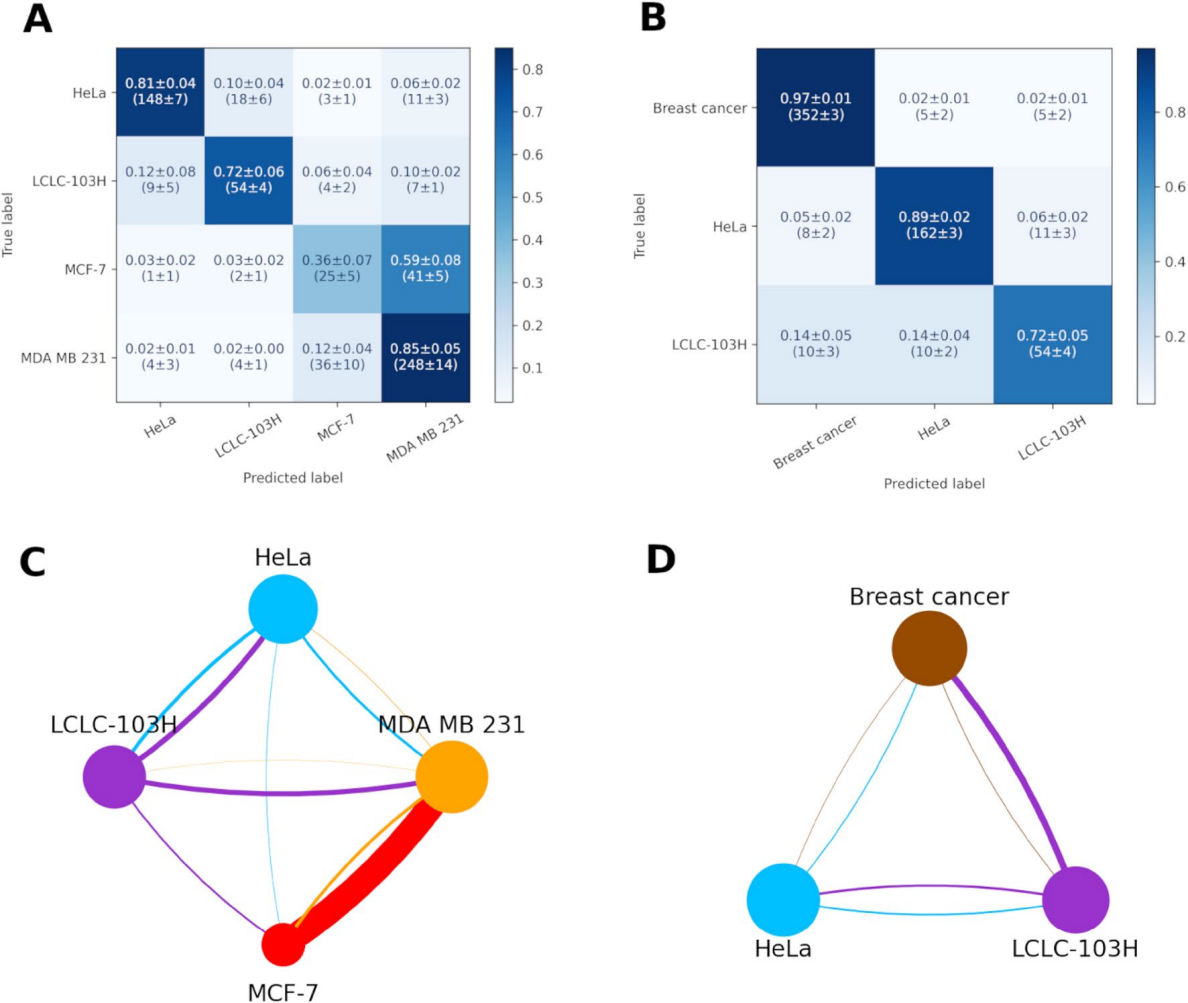
Confusion matrixes calculated from the test datasets of the cross-validations (**A**,**B**) and graphs (MCDCNN, 120-min) (**C**,**D**) of the four cancer cell lines MDA-MB-231, MCF-7, HeLa and LCLC-103H. First, the two breast cancer cell lines were treated separately and then considered as a single group.

The first confusion matrix and confusion graph (Fig. 4A,C) shows that with the addition of the two other cell lines, the distinction between the two breast cancers deteriorated a little, the true positive rate of MCF-7 decreased from 39 to 36%, however, the true positive rate of MDA-MB-231 increased a little from 87 to 85%. The neural network classifies 51% of the MCF-7 cells as MDA-MB-231. There are also two relatively big false positive rates in the case of LCLC-103H cells classified as HeLa (12%) or MDA-MB-231 (10%) cells.

To improve the problem as mentioned earlier with the breast cancer cell lines, we tried the classification with these two lines considered as one group. The accuracy increased significantly from (72** ± **1)% to (91** ± **1)% (Table 3). As shown on the second confusion matrix and confusion graph (Fig. 4B,D), the true positive rate increased in the case of breast cancer (36%, 85 to > 97%) and Hela cells (81 to > 89%) and it didn’t change in the case of LCLC-103H cells (72 to > 72%).

**Figure 5:**
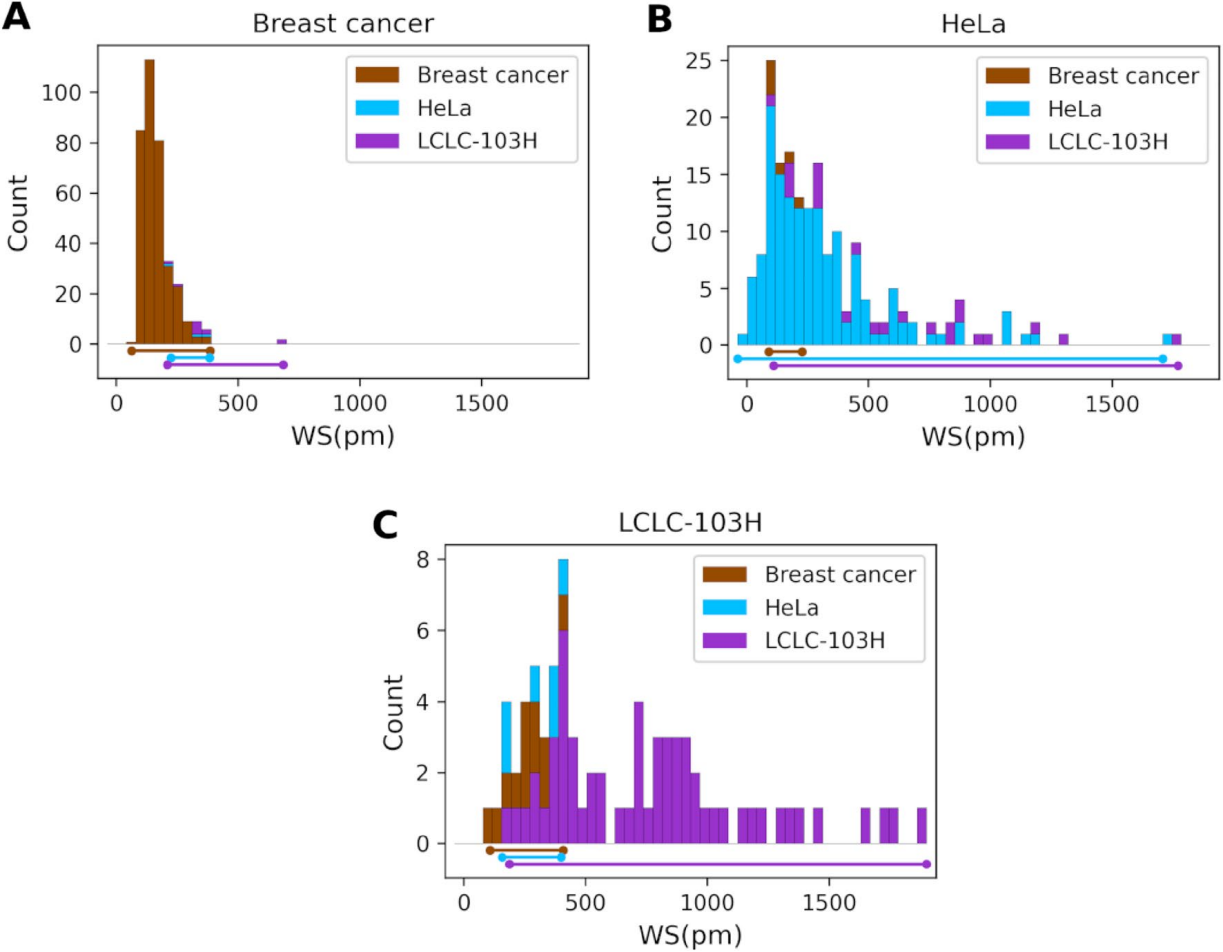
Adhesion signal distributions in wavelength shift depicting the false negative errors (MCDCNN, 120-min) from one iteration of the cross-validations for the four cancer cell lines breast cancer (MDA-MB-231, MCF-7) (**A**), HeLa (**B**) and LCLC-103H (**C**).

To visualize where the neural network misclassifies, we plotted the adhesion signal distribution (Fig. 5). In the case of breast cancer (Fig. 5A) there are false negatives equally from HeLa and LCLC-103H cells with only LCLC-103H false negatives appearing in the 400–700 pm range and both LCLC-103H and HeLa false negatives appearing in the 200–400 pm range, only in the very low adhesion region (0–200 pm) there aren’t any false negatives.

In the case of LCLC-103H cells (Fig. 5C) and HeLa cells (Fig. 5B) breast cancer false negatives dominate the low adhesion region (50–450 pm). LCLC-103H false negative errors appear in the entire range of HeLa signals (100–1800 pm) (Fig. 5B) same as HeLa false negatives in the LCLC-103H signals (Fig. 5C).

### All measured cell types

**Figure 6:**
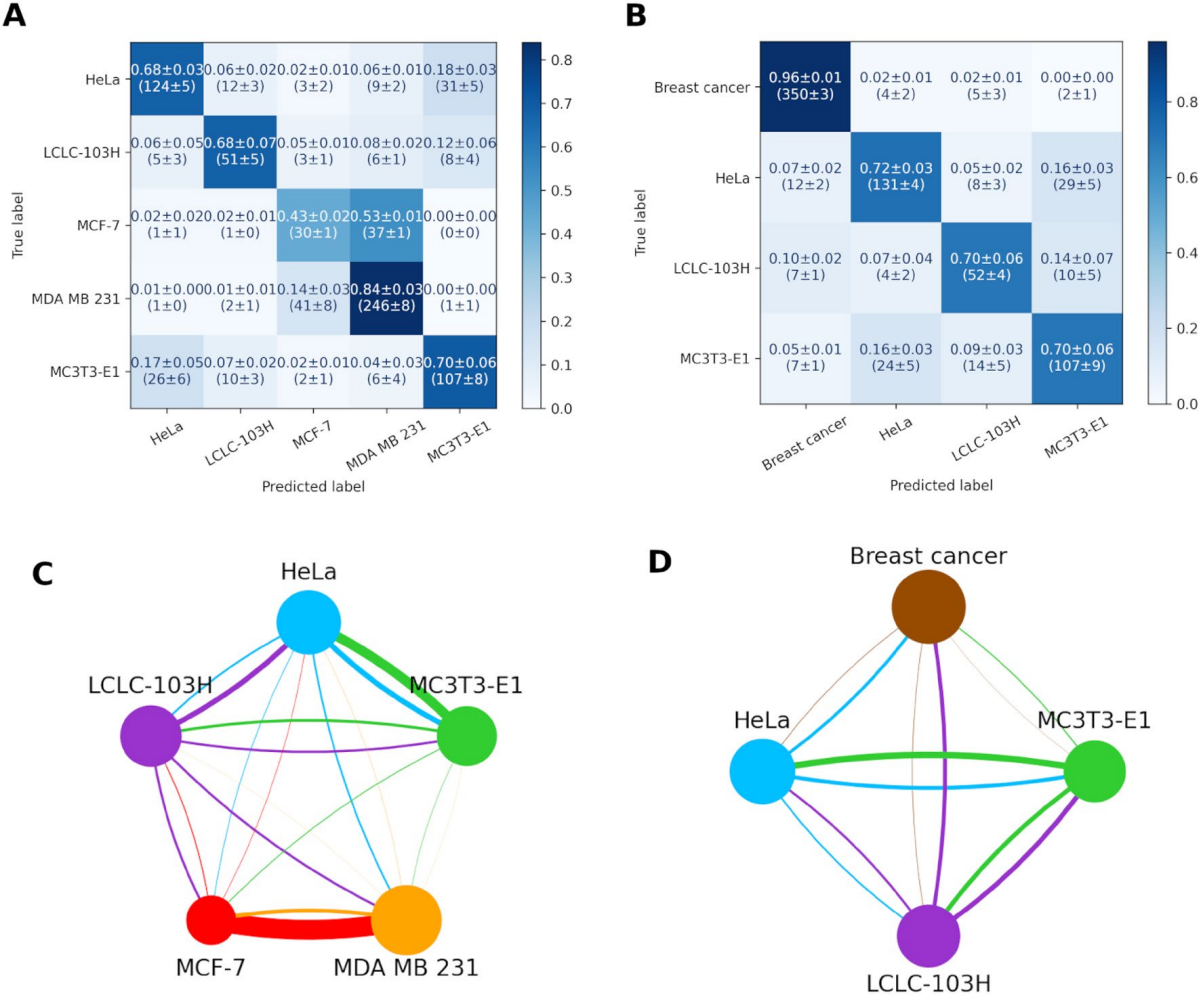
Confusion matrixes calculated from the test datasets of the cross-validations (**A**,**B**) and graphs (MCDCNN, 150-min) (**C**,**D**) of all the measured cell lines: MDA-MB-231, MCF-7, HeLa, LCLC-103H and MC3T3-E1. First, the two breast cancer cell lines (MDA-MB231 and MCF-7) were treated separately and then considered as a single group.

In the case of all measured cell types, the two breast cancer cell lines have the most significant negative (53%) error ratio (Fig. 6). As shown in the previous case we also combined these two cell lines into one group to increase the accuracy (from (65** ± **1) to (83** ± **1)%). It significantly increased the true positive rates from 68 to 72% (HeLa) and from 43 and 84% to 96% (breast cancer) and from 68 to 70% (LCLC-103H) and didn’t changed in the case of MC3T3-E1 (70%) (see Fig. 6).

The difference in organism, tissue, cell type or disease (Table 4) did not seem to affect the true positive rates of the classification, only the two breast cancer cell types were easily confused with each other by the classifier.

**Table 4:** Summary of the evaluated cell lines.

Name	Organism		Tissue	Cell type	Disease
HeLa	Human	Cancer	Cervix	Epithelial	Adenocarcinoma
LCLC-103H	Human	Cancer	Lung		Large cell carcinoma
MC3T3-E1	Mouse	–	Calvaria	Osteoblastic	–
MCF-7	Human	Cancer	Breast	Epithelial	Adenocarcinoma
MDA-MB-231	Human	Cancer	Breast	Epithelial	Adenocarcinoma

**Figure 7:**
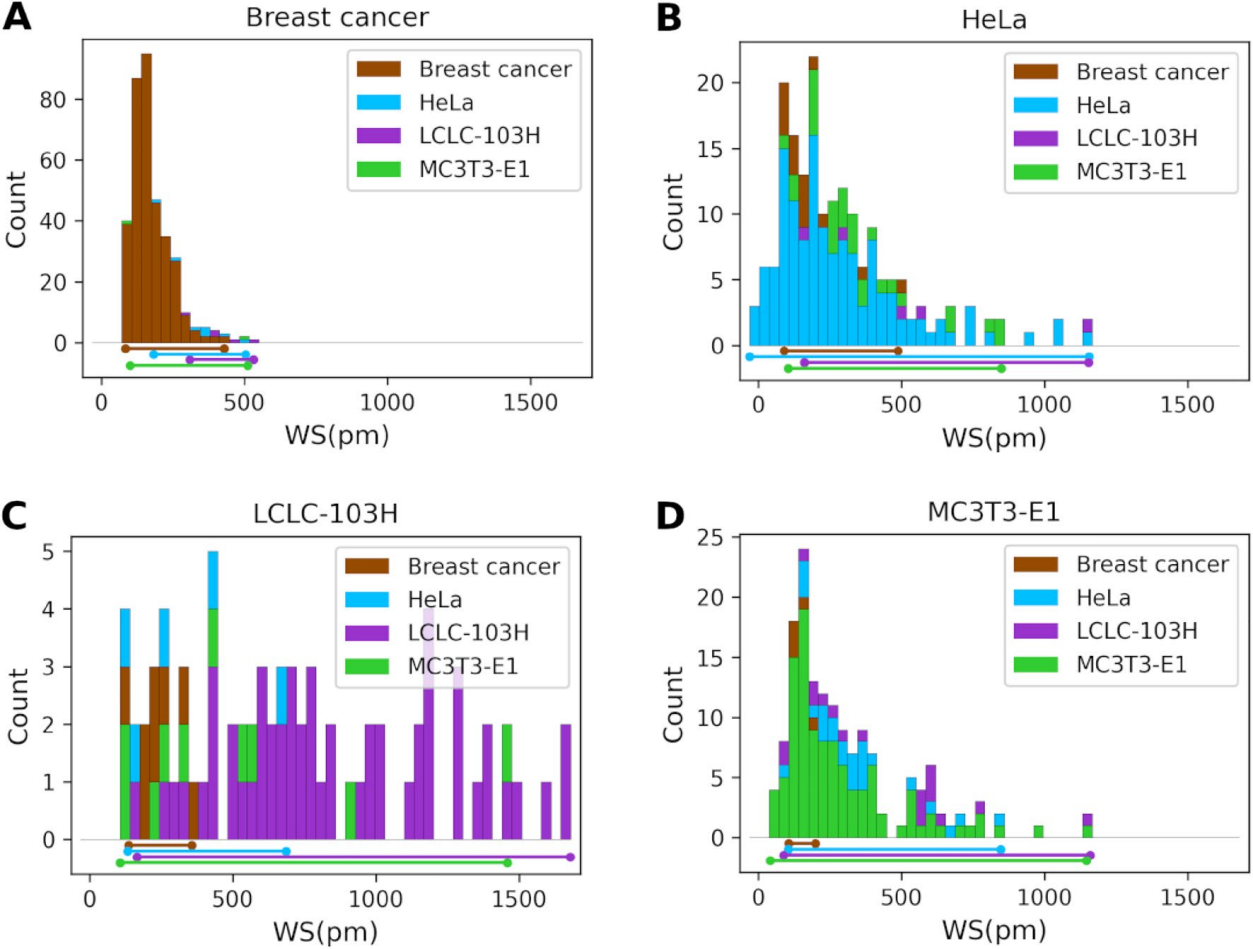
Wavelength shift distributions depicting the false negative errors (MCDCNN, 150-min) from one iteration of the cross-validations for all the measured cell lines: breast cancer (MDA-MB-231, MCF-7) (**A**), HeLa (**B**), LCLC-103H (**C**) and MC3T3-E1 (**D**).

Breast cancer false negatives dominate the low adhesion region (100–500 pm) (Fig. 7B–D), while LCLC-103H (Fig. 7A,B,D) and MC3T3-E1 (Fig. 7A–C) false negatives are present in the whole adhesion region (100–1450 pm) and HeLa false negatives are present in the low-medium adhesion region (100–820 pm) (Fig. 7A,C,D).

For diagnostic purposes, it should be better to use the combined breast cancer class for training and classification as it gave higher accuracy in the case of all cell types excluding MC3T3-E1 where the accuracy didn’t change identification.

**Figure 8:**
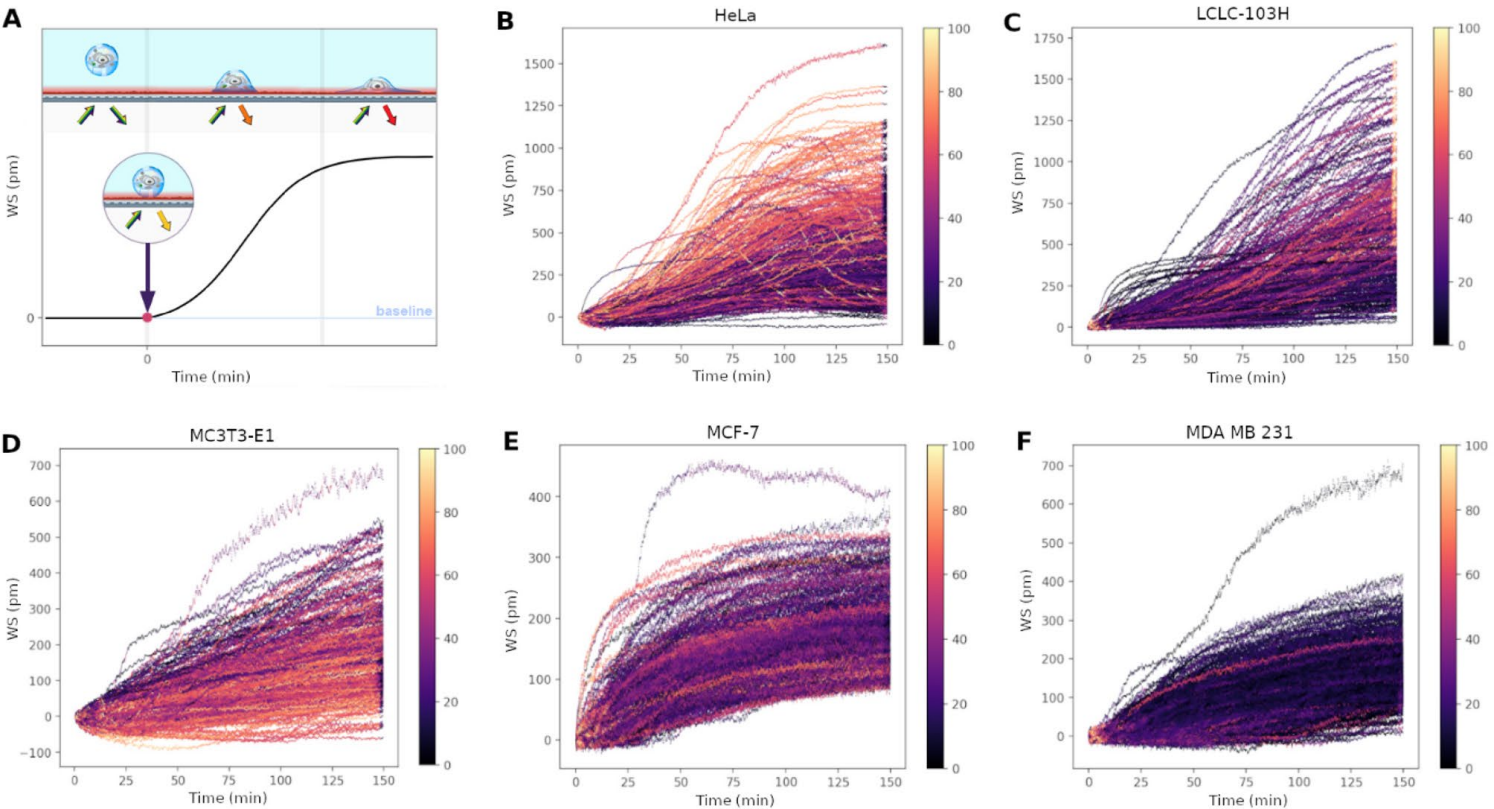
Schematic illustration of the cell adhesion during the single-cell biosensor measurements (**A**) and grad-CAM visualization of single-cell signals: Hela (**B**), LCLC-103H (**C**), MC3T3-E1 (**D**), MCF-7 (**E**) and MDA-MB-231 (**F**).

To analyze the differences between the cell types we used grad-CAM to visualize what were essential features for neural network during the classification task (Fig. 8B–F). For easier understanding we also made an illustration about the cell adhesion phases corresponding to the single-cell biosensor signal (Fig. 8A). Based on Fig. 8 we can assume that during classification high adhesion signals are characteristic of Hela cells, low adhesion signals are typical of MC3T3-E1 cells. For LCLC-103H cells, the neural network decides based on the end-time distribution of the adhesion signals, for MCF-7 cells we assume the shape of the adhesion signals are the most important especially in the early phases of adhesion. We presume that MDA-MB-231 cells didn’t have any unique traits, so the neural network assigned the ‘leftover’ adhesion phase, the very start of the measurement.

## Conclusions

Our goal was to investigate the possibility of utilizing adhesion kinetic signal-based single-cell classification. A database was created with more than 4500 single-cell label-free optical biosensor signals, comprising different cell type measurements in equal amount. This dataset was then used as a benchmark to train a wide range of task-specific time-series classifier models that can identify cell types solely based on their biosensor-recorded adhesion kinetics. Both traditional classifiers and deep learning methods were proposed for the problem and tested. Notably, the deep learning models (after finding optimal architectures) resulted in a classification accuracy of 64–91%, depending on the classification task and neural network. A possible direction for improvement would be to not restrict the dataset to the single maximal pixel values of adherent cells but instead, use the temporal sequence of 2D sensor images as inputs for the CNN classifier. As these images contain rich spatial information on the cell adhesion process, it is expected to increase significantly the performance.

Aspiring to give biological interpretation to these results, we plotted the adhesion signal distribution depicting the false negative hits. In all cases, the breast cancer false negatives dominated the low adhesion region, while the other cell line false negatives were basically present in the whole adhesion region.

The introduced label-free kinetic method is noninvasive and does not affect cellular functionality. Therefore, it could also be adapted for applications where the selected cells have to be processed further, such as immune therapy and regenerative medicine.

## Data Availability

The data handling and cell analysis software code is available at https://github.com/Nanobiosensorics/single-cell-tsc. The analyzed label-free dataset can be downloaded from https://nc.ek-cer.hu/index.php/s/wqE3LHxZdCbsngz.

## References

[1] Alberts, B. *et al. Molecular Biology of the Cell*. *Molecular Biology of the Cell* (Second edition. New York: Garland Pub., [1989] ©1989, 2017). 10.1201/9781315735368.

[2] Giancotti, F. G. & Ruoslahti, E. Integrin Signaling. *Science (80-. ).* **285**, 1028–1033 (1999). 10.1126/science.285.5430.1028 10446041

[3] Hynes RO (2002). Integrins: bidirectional, allosteric signaling machines. Cell.

[4] Bianconi MatthiasAU - Prager, Gerald W.TI - Integrins in the Spotlight of Cancer, D.-U. No Title. *Int. J. Mol. Sci*. **17** (2016). 10.3390/ijms17122037 PMC5187837 27929432

[5] Lodish, H. *et al. Molecular cell biology*. (Macmillan, 2008).

[6] Kanyo, N. *et al.* Glycocalyx regulates the strength and kinetics of cancer cell adhesion revealed by biophysical models based on high-resolution label-free optical data. *Sci. Rep.* **10**, (2020). 10.1038/s41598-020-80033-6 PMC7773743 33380731

[7] Janiszewska M, Primi MC, Izard T (2020). Cell adhesion in cancer: Beyond the migration of single cells. J. Biol. Chem..

[8] Gawad C, Koh W, Quake SR (2016). Single-cell genome sequencing: Current state of the science. Nat. Rev. Genet..

[9] Lee J, Hyeon DY, Hwang D (2020). Single-cell multiomics: technologies and data analysis methods. Exp. Mol. Med..

[10] Chen W (2022). Live-seq enables temporal transcriptomic recording of single cells. Nature.

[11] Sztilkovics M (2020). Single-cell adhesion force kinetics of cell populations from combined label-free optical biosensor and robotic fluidic force microscopy. Sci. Rep..

[12] Schlotter T (2020). Force-controlled formation of dynamic nanopores for single-biomolecule sensing and single-cell secretomics. ACS Nano.

[13] Danielsson B, Hedberg U, Rank M, Xie B (1992). Recent investigations on calorimetric biosensors. Sens. Actuators B Chem..

[14] Karunakaran, C., Rajkumar, R. & Bhargava, K. Introduction to biosensors. In *Biosensors and bioelectronics* 1–68 (Elsevier, 2015).

[15] Pohanka, M. & Skládal, P. Electrochemical biosensors—principles and applications. *J. Appl. Biomed.* **6**, (2008).

[16] Hammond JL, Formisano N, Estrela P, Carrara S, Tkac J (2016). Electrochemical biosensors and nanobiosensors. Essays Biochem..

[17] Skládal P (2016). Piezoelectric biosensors. TrAC Trends Anal. Chem..

[18] Pohanka M (2017). The piezoelectric biosensors: Principles and applications. Int. J. Electrochem. Sci.

[19] Ligler, F. S. & Taitt, C. R. *Optical biosensors: present & future*. (Gulf Professional Publishing, 2002).

[20] Ramsden JJ (1997). Optical biosensors. J. Mol. Recognit..

[21] Fang Y (2007). Non-invasive optical biosensor for probing cell signaling. Sensors.

[22] Saftics A (2021). Data evaluation for surface-sensitive label-free methods to obtain real-time kinetic and structural information of thin films: A practical review with related software packages. Adv. Colloid Interface Sci..

[23] Fang Y (2010). Live cell optical sensing for high throughput applications. Adv. Biochem. Eng. Biotechnol..

[24] Fang Y (2006). Label-free cell-based assays with optical biosensors in drug discovery. Assay Drug Dev. Technol..

[25] Orgovan N (2014). Dependence of cancer cell adhesion kinetics on integrin ligand surface density measured by a high-throughput label-free resonant waveguide grating biosensor. Sci. Rep..

[26] Szekacs I, Orgovan N, Peter B, Kovacs B, Horvath R (2018). Receptor specific adhesion assay for the quantification of integrin–ligand interactions in intact cells using a microplate based, label-free optical biosensor. Sensors Act. B Chem..

[27] Szekacs, I. *et al.* Integrin targeting of glyphosate and its cell adhesion modulation effects on osteoblastic MC3T3-E1 cells revealed by label-free optical biosensing. *Sci. Rep.* **8**, (2018). 10.1038/s41598-018-36081-0 PMC6258691 30479368

[28] Bagnall A, Lines J, Bostrom A, Large J, Keogh E (2017). The great time series classification bake off: A review and experimental evaluation of recent algorithmic advances. Data Min. Knowl. Discov..

[29] Potes, C., Parvaneh, S., Rahman, A. & Conroy, B. Ensemble of feature-based and deep learning-based classifiers for detection of abnormal heart sounds. In *2016 computing in cardiology conference (CinC)* 621–624 (IEEE, 2016).

[30] Das, V. & Mukerji, T. Traditional feature based vs direct machine learning based AVO classification. In *81st EAGE Conference and Exhibition 2019* vol. 2019 1–5 (European Association of Geoscientists & Engineers, 2019).

[31] Lines, J., Taylor, S. & Bagnall, A. Time series classification with HIVE-COTE: The hierarchical vote collective of transformation-based ensembles. *ACM Trans. Knowl. Discov. Data* **12**, (2018).

[32] Barredo Arrieta, A. *et al.* Explainable Artificial Intelligence (XAI): Concepts, taxonomies, opportunities and challenges toward responsible AI. *Inf. Fusion* **58**, 82–115 (2020).

[33] Selvaraju, R. R. *et al.* Grad-cam: Visual explanations from deep networks via gradient-based localization. In *Proceedings of the IEEE international conference on computer vision* 618–626 (2017).

[34] Erhan, D., Courville, A. & Bengio, Y. Understanding representations learned in deep architectures. (2010).

[35] Vianay B (2018). Variation in traction forces during cell cycle progression. Biol. Cell.

[36] Kurzawa L, Morris MC (2010). Cell-cycle markers and biosensors. Chembiochem.

[37] Margadant C, van Opstal A, Boonstra J (2007). Focal adhesion signaling and actin stress fibers are dispensable for progression through the ongoing cell cycle. J. Cell Sci..

[38] Lundgren E, Roos G (1976). Cell surface changes in HeLa cells as an indication of cell cycle events. Cancer Res..

[39] Jones MC, Askari JA, Humphries JD, Humphries MJ (2018). Cell adhesion is regulated by CDK1 during the cell cycle. J. Cell Biol..

[40] Moes MJA, Bijvelt JJ, Boonstra J (2011). Attachment of HeLa cells during early G1 phase. Histochem. Cell Biol..

[41] Fawaz, H. I., Forestier, G., Weber, J., Idoumghar, L., & Muller, P. A. Deep learning for time series classification: A review—data mining and knowledge discovery. SpringerLink, March 2 (2019).

[42] Chawla NV, Bowyer KW, Hall LO, Kegelmeyer WP (2002). SMOTE: Synthetic minority over-sampling technique. J. Artif. Intell. Res..

[43] Lemaitre G, Nogueira F, Aridas CK (2017). Imbalanced-learn: A python toolbox to tackle the curse of imbalanced datasets in machine learning. J. Mach. Learn. Res..

[44] Pedregosa F, Varoquaux G, Gramfort A, Michel V, Thirion B, Grisel O, Blondel M, Prettenhofer P, Weiss R, Dubourg V, Vanderplas J, Passos A, Cournapeau D, Brucher M, Perrot M, Duchesnay E (2011). Scikit-learn: Machine learning in python. J. Mach. Learn. Res..

[45] Ismail Fawaz H, Lucas B, Forestier G (2020). InceptionTime: Finding AlexNet for time series classification. Data Min. Knowl. Disc..

[46] Zheng, Y., Liu, Q., Chen, E., Ge, Y., & Zhao, J. L. Time series classification using multi-channels deep convolutional neural networks. In: *Web-age information management*, pp 298–310 (2014).

[47] Chollet, F., *et al.* Keras (2015).

[48] Zhang H, Huang L, Wu CQ, Li Z (2020). An effective convolutional neural network based on SMOTE and Gaussian mixture model for intrusion detection in imbalanced dataset. Comput. Netw..

[49] D’Orazio M, Murdocca M, Mencattini A, Casti P, Filippi J, Antonelli G, di Giuseppe D, Comes MC, di Natale C, Sangiuolo F, Martinelli E (2022). Machine learning phenomics (MLP) combining deep learning with time-lapse-microscopy for monitoring colorectal adenocarcinoma cells gene expression and drug-response. Sci. Rep..

[50] D’Orazio M, Corsi F, Mencattini A, di Giuseppe D, Colomba Comes M, Casti P, Filippi J, di Natale C, Ghibelli L, Martinelli E (2020). Deciphering cancer cell behavior from motility and shape features: Peer prediction and dynamic selection to support cancer diagnosis and therapy. Front. Oncol..

[51] Filippi J, di Giuseppe D, Casti P, Mencattini A, Antonelli G, D’Orazio M, Corsi F, Della-Morte Canosci D, Ghibelli L, Witte C, di Natale C, Neale SL, Martinelli E (2022). Exploiting spectral information in Opto-Electronic Tweezers for cell classification and drug response evaluation. Sens. Actuators B Chem..

[52] Mencattini A, D’Orazio M, Casti P, Comes MC, di Giuseppe D, Antonelli G, Filippi J, Corsi F, Ghibelli L, Veith I, di Natale C, Parrini MC, Martinelli E (2023). Deep-Manager: A versatile tool for optimal feature selection in live-cell imaging analysis. Commun. Biol..

[53] Comes MC, Filippi J, Mencattini A, Corsi F, Casti P, de Ninno A, di Giuseppe D, D’Orazio M, Ghibelli L, Mattei F, Schiavoni G, Businaro L, di Natale C, Martinelli E (2020). Accelerating the experimental responses on cell behaviors: A long-term prediction of cell trajectories using Social Generative Adversarial Network. Sci. Rep..

[54] Comes MC, Mencattini A, di Giuseppe D, Filippi J, D’orazio M, Casti P, Corsi F, Ghibelli L, di Natale C, Martinelli E (2020). A camera sensors-based system to study drug effects on in vitro motility: The case of PC-3 prostate cancer cells. Sensors (Switzerland).

